# Obesity- and lipid-related indices as a predictor of type 2 diabetes in a national cohort study

**DOI:** 10.3389/fendo.2023.1331739

**Published:** 2024-01-31

**Authors:** Ying Wang, Xiaoyun Zhang, Yuqing Li, Jiaofeng Gui, Yujin Mei, Xue Yang, Haiyang Liu, Lei-lei Guo, Jinlong Li, Yunxiao Lei, Xiaoping Li, Lu Sun, Liu Yang, Ting Yuan, Congzhi Wang, Dongmei Zhang, Jing Li, Mingming Liu, Ying Hua, Lin Zhang

**Affiliations:** ^1^ Department of Graduate School, Wannan Medical College, Wuhu, An Hui, China; ^2^ Student Health Center, Wannan Medical College, Wuhu, An Hui, China; ^3^ Department of Surgical Nursing, School of Nursing, Jinzhou Medical University, Linghe District, Jinzhou, Liaoning, China; ^4^ Department of Occupational and Environmental Health, Key Laboratory of Occupational Health and Safety for Coal Industry in Hebei Province, School of Public Health, North China University of Science and Technology, Tangshan, Hebei, China; ^5^ Obstetrics and Gynecology Nursing, School of Nursing, Wannan Medical College, Wuhu, An Hui, China; ^6^ Department of Emergency and Critical Care Nursing, School of Nursing, Wannan Medical College, Wuhu, An Hui, China; ^7^ Department of Internal Medicine Nursing, School of Nursing, Wannan Medical College, Wuhu, An Hui, China; ^8^ Department of Pediatric Nursing, School of Nursing, Wannan Medical College, Wuhu, An Hui, China; ^9^ Department of Surgical Nursing, School of Nursing, Wannan Medical College, 22 Wenchang West Road, Higher Education Park, Wuhu, An Hui, China; ^10^ Rehabilitation Nursing, School of Nursing, Wanna Medical College, 22 Wenchang West Road, Higher Education Park, Wuhu, An Hui, China

**Keywords:** type 2 diabetes, National cohort study, obesity, middle-aged and elderly, receiver operating characteristic curve

## Abstract

**Objective:**

Type 2 diabetes mellitus (T2DM) remains a major and widespread public health concern throughout the world. The prevalence of T2DM in the elderly has risen to the top of the list of public health concerns. In this study, obesity- and lipid-related indices were used to predict T2DM in middle-aged and elderly Chinese adults.

**Methods:**

The data came from the China Health and Retirement Longitudinal Study (CHARLS), including 7902 middle-aged and elderly participants aged 45 years or above. The study assessed the association of obesity- and lipid-related indices and T2DM by measuring 13 indicators, including body mass index (BMI), waist circumference(WC), waist-height ratio (WHtR), conicity index(CI), visceral adiposity index (VAI), Chinese visceral adiposity index (CVAI), lipid accumulation product (LAP), a body shape index (ABSI), body roundness index (BRI), triglyceride glucose index (TyG-index) and its correlation index (TyG-BMI, TyG-WC, TyG-WHtR). The association of 13 obesity- and lipid-related indices with T2DM was investigated by binary logistic regression. Additionally, the predictive anthropometric index was evaluated, and the ideal cut-off value was established using the receiver operating characteristic (ROC) curve analysis and area under the curve (AUC).

**Results:**

The study included 7902 participants, of whom 3638(46.04) and 4264(53.96) were male and female. The prevalence of T2DM in mid-aged and old adults in China was 9.02% in males and 9.15% in females. All the above 13 indicators show a modest predictive power (AUC>0.5), which was significant for predicting T2DM in adults (middle-aged and elderly people) in China (*P*<0.05). The results revealed that TyG-WHtR [AUC =0.600, 95%CI: 0.566–0.634] in males and in females [AUC =0.664, 95%CI: 0.636–0.691] was the best predictor of T2DM (*P*<0.05).

**Conclusion:**

Most obesity- and lipid-related indices have important value in predicting T2DM. Our results can provide measures for the early identification of T2DM in mid-aged and elderly Chinese to reduce the prevalence of T2DM and improve health.

## Introduction

The percentage of older adults (defined as those 60 years of age or older) is expected to increase and reach >20% by the year 2050. China has the world’s largest elderly population, 201 million in 2015, which is expected to rise to 479 million by 2050 (1). Type 2 diabetes mellitus(T2DM) is a worldwide problem and the most prevalent metabolic disease affecting about 1 in 10 adults (10.5% of adults worldwide) (2). Over the last two years, the prevalence of T2DM has increased by 16%, which is an alarming increase (2). Without taking into account the risk of death associated with COVID-19, in 2021, approximately 12.2% of adult all-cause deaths worldwide will be due to diabetes and its complications (3). In addition, 10.6% of adults in the world have poor glucose tolerance, which puts them at high risk of developing T2DM (3). In parallel with the rise in prevalence, the economic costs of T2DM have also increased dramatically. Controlling the incidence of T2DM is the key to reducing the cost of global economic development and improving the health of individuals and communities.

T2DM is associated with a variety of factors. The increase in the incidence of T2DM is mainly due to changes in living conditions and habits, such as a sedentary lifestyle (4), physical inactivity (5), smoking (6), and alcohol consumption (7). Several epidemiological investigations have shown that obesity is the most important risk factor for T2DM, and it has a certain impact on the production of insulin resistance and disease progression (8). The epidemic of obesity contributes to the burden of T2DM in worldwide. In a meta-analysis of Mendelian randomization studies, the easiest and most generally used indicator of obesity, body mass index (BMI), was linked to a higher risk of T2DM (9). However, BMI is not entirely inclusive. Such as WC (waist circumference), waist-height ratio (WHtR), and lipid accumulation index (LAP) have been widely used in epidemiology to measure obesity or central obesity (10–13). We can use these indicators to explore the risk of developing T2DM.

It should be noted that no agreement has been reached on the association of obesity and lipid-associated indices with T2DM. For instance, the BMI is frequently used to estimate the likelihood of contracting chronic diseases including diabetes, hypertension, depression, and cancer (14–17). However, compared to other measurements like WC, WHR, and WHtR, BMI was the least accurate predictor for cardiovascular risk factors such as diabetes, hypertension, and dyslipidemia in the study conducted by Lee et al. (18). Some studies have suggested that WC appears to be a stronger predictor of diabetes than BMI in Western populations (19, 20), while the evidence on this remains contradictory in Asian populations (21, 22). Previous studies reported that the Chinese visceral adiposity index (CVAI) was superior to BMI, WC, or visceral adiposity index (VAI) for the diagnosis of diabetes and prediabetes (23, 24). Another study in Japan found that triglyceride glucose index (TyG-index) was linearly associated with the risk of T2DM in the population (25). From these studies, it can be concluded that obesity and lipid-related indices are closely related to T2DM. However, it is not clear which index performs better in predicting diabetes risk. Moreover, most of the research is carried out based on the West, and the results are different because of the differences in nationality and culture. Therefore, it is of great significance to further determine the correlation and predictive value between obesity and lipid-related indicators and T2DM in middle-aged and elderly people for guiding the prevention and treatment of T2DM in elderly people.

Currently, only a small number of cross-sectional studies have investigated the relationship between obesity and T2DM in elderly Chinese adults, but few studies have explored multiple indicators to predict T2DM risk, so to fill these gaps, we conducted a national retrospective cohort study in older Chinese adults. The longitudinal effects of 13 indicators on the incidence of T2DM were examined to predict the incidence of T2DM. This study provides a theoretical basis for early detection of T2DM, advanced and scientific medical prevention, and avoiding the occurrence of complications of T2DM, significantly improving the living standards of the elderly and prolonging life.

## Method

### Study design and setting

For this national cohort study, we used data from the China Wave2011 Longitudinal Study of Health and Retirement, a national longitudinal study of middle-aged and older Chinese people and their spouses, who were evaluated at baseline and followed up for two years (26). Individuals without T2DM in baseline from the China Health and Retirement Longitudinal Study (CHARLS) Wave 2011 study were included in our analysis after excluding subjects with missing data. Data gathering was then carried out in 2015. We enrolled 7902 individuals over 45 years of age in our nationwide cohort. The CHARLS project received approval from Peking University’s Biomedical Ethics Review Board, and each participant signed an informed consent form. The data and relevant data for this study are available at the CHARLS project website (http://charls.pku.edu.cn/).

### Individuals

The subjects of this survey were selected from the CHARLS, Wave 1 (2011). The CHARLS Wave 2011 was used to select participants. Cohort studies are used in this investigation. In 2011, patients with undiagnosed T2DM were included in our follow-up cohort. Four years later, in 2015, the incident rate of those affected by 13 indicators was assessed.

### Baseline characteristics

At baseline, trained researchers used a structured questionnaire to acquire sociodemographic status information and health-related indicators, including age, education, marriage, residence, drinking, smoking, activities, exercise, and chronic diseases. The majority of factors were based on our earlier research investigations (27–32).

### Data collection and definitions

Fasting plasma glucose (FPG), HbA1c, TC, triglycerides (TG), high-density lipoprotein-cholesterol (HDL-c), and low-density lipoprotein-cholesterol (LDL-c) were measured by fasting blood samples. FPG, TG, TC, HDL-c, and LDL-c were determined by enzyme colorimetric assay, while the HbA1c test was carried out by high-performance liquid chromatography (HPLC) (33). T2DM was defined as *1*) self-reported doctor-diagnosed diabetes, *2*) fasting plasma glucose ≥7.0mmol/L, *3*) 2-h plasma glucose ≥11.1mmol/L, or *4*) HbA1c ≥6.5% (34). Participants with a diagnosis of T2DM at baseline were excluded from this analysis.

### Measurements

The BMI was calculated based on the weight and height of the participants. According to the Chinese standard, BMI was classified into three categories: obesity (BMI ≥ 28 kg/m2), overweight (24 ≤ BMI< 28 kg/m2), and underweight and normal (BMI< 24 kg/m2) (35). The lower rib margin and the middle of the flexible measuring tape were used to determine the WC (32). Conicity Index (CI) was measured by WC, weight, and height (36). When calculating the Body shape Index (BSI), body roundness index (BRI), and VAI, it should be noted that VAI is not gender-specific like other measurements (37–39). WHtR is defined as the WC (m) divided by the height (m) (40). The LAP is calculated somewhat differently by subtracting from the WC (men: 65 cm, women: 58 cm) and multiplying it with TG (41). In order to create a more suitable metric for Chinese people, CVAI is built on VAI (42). The following formulas were used to measure the other anthropometric indexes (Equations 1-12).


(1) $$BMI=\frac{Weight}{Height^{2}}$$

(2) $$WHtR=\frac{WC}{Height}$$

(3) $$\text{Males}:VAI=\frac{WC}{39.68+(1.88\times BMI)}\times \frac{TG}{0.81}\times \frac{1.52}{HDL}$$

$$\text{Females}:VAI=\frac{WC}{36.58+(1.89\times BMI)}\times \frac{TG}{0.81}\times \frac{1.52}{HDL}$$

(4) $$ABSI=\frac{WC}{Height^{\frac{1}{2}}\times BMI^{\frac{2}{3}}}$$

(5) $$\text{BRI}=364.2-365.5\sqrt{1-\left(\frac{WC÷{\left(2\pi \right)}^{2}}{{\left(0.5\times Height\right)}^{2}}\right)}$$

(6) Males: LAP= [WC (cm)−65] ×TG (mmol/l)

Females: LAP= [WC (cm)−58] ×TG (mmol/l)

(7) $$CI=\frac{WC(m)}{0.019\sqrt{\frac{weight(kg)}{height(m)}}}$$

(8) $$\text{Males}:CVAI=-267.93+0.68\times age+0.03\times BMI\;\left(kg/m2\right)\;+4.00\times WC\;\left(cm\right)+22.00\times Log_{10}TG\;\left(mmol/l\right)-16.32\times HDL-C\left(mmol/l\right)$$

$$\text{Females}:CVAI=-187.32+1.71\times age+4.32\times BMI\;\left(kg/m2\right)\;+1.12\times WC\;\left(cm\right)+39.76\times Log_{10}TG\;\left(mmol/l\right)-11.66\times HDL-C\left(mmol/l\right)$$

(9) TyG index=Ln[(TG(mg/dl)×glucose(mg/dl)/2)]

(10) TyG−BMI= TyG×BMI

(11) TyG−WC= TyG×WC

(12) TyG −WHtR= TyG×WHtR

### Statistical analysis

All the statistical analyses were analyzed using STATA software version 25.0. Continuous variables were applied by mean (standard deviation, SD) depending on normal distribution or not, while the frequency with percentage was presented for categorical variables. A chi-squared test was run to compare dichotomous or categorical variables. Results from studies that were gender-specific are also included because the relationship between T2DM and obesity status varies between the sexes. The odds ratios (OR) and 95% confidence intervals (CI) for each of the 13 obesity- and lipid-related indices were calculated by binary logistic regression after adjusting for age, education, marital status, current residence, current smoking, alcohol consumption, activity participation, regular exercise, and chronic disease. The 13 indices were divided into two categories based on their optimal cutoff values. The P-value obtained by the statistical significance test is usually regarded as the statistical significance when *P*< 0.05, and the *P*< 0.001 is very significant. The ability of these indices to distinguish T2DM was tested by drawing the receiver operating curve (ROC curve) and AUC calculation. The ROC curve can be used to calculate the sensitivity, specificity, positive, negative, positive, positive, and negative. A Youden index (sensitivity + specificity 1) was used to select the cut-off points to evaluate classification accuracy.

## Results

**Table 1** shows the characteristics of the participants with complete samples. This study included 7902 patients, of which 3638 (46. 04%) were male, and 4 264 (53.96%) were female. Most of them were 45-64 years old, there were significant differences between men and women in age, education, marital status, current smoking, alcohol drinking, number of chronic diseases, WC, BMI, WHtR, VAI, ABSI, BRI, LAP, CI, CVAI, TyG index, TyG-BMI, TyG-WC and TyG-WHtR (*P<* 0.05). However, there was no statistical significance in the distribution of current residence, taking activities, and having regular exercises between the two groups (*P* > 0.05). Due to these marked gender differences (*P*< 0.05), the main analysis was conducted separately by gender.

**Table 1:** Characteristics of participants with full samples(N=7902).

Variables	Male	Female	Total	*t/χ^2^*	*P*
	N (%)	N (%)	N (%)		
N	3638 (46.04)	4264 (53.96)	7902 (100)		
Age(years)					
45-54	1107 (30.43)	167 3(39.24)	2780 (35.18)	79.602	< 0.001
55-64	1420 (39.03)	1562 (36.63)	2982 (37.74)		
65-74	814 (22.37)	716 (16.79)	1530 (19.36)		
≥75	297 (8.16)	313 (7.34)	610 (7.72)		
Education					
Illiterate	501 (13.77)	1807 (42.38)	2308 (29.21)	796.902	< 0.001
Less than elementary school	2669 (73.36)	2173 (50.96)	4842 (61.28)		
High school	306 (8.41)	212 (4.97)	518 (6.56)		
Above vocational school	162 (4.45)	72 (1.69)	234(2.96)		
Marital status					
Single	337(9.26)	645 (15.13)	982 (12.43)	62.013	< 0.001
Married	3301(90.74)	3619 (84.87)	6920 (87.57)		
Current residence					
Rural	3363(92.44)	3968 (93.06)	7331 (92.77)	1.116	0.291
Urban	275(7.56)	296 (6.94)	571 (7.23)		
Current smoking					
No	893(24.55)	3937 (92.33)	4830 (61.12)	3796.205	< 0.001
Former smoke	585(16.08)	77 (1.81)	662(8.38)		
Current smoke	2160(59.37)	250 (5.86)	2410 (30.50)		
Alcohol drinking					
No	1592(43.76)	3730 (87.48)	5322 (67.35)	1771.074	< 0.001
Less than once a month	403(11.08)	215 (5.04)	618 (7.82)		
More than once a month	1643(45.16)	319 (7.48)	1962 (24.83)		
Taking activities					
No	1801(49.51)	2161 (50.68)	3962 (50.14)	1.084	0.298
Yes	1837(50.49)	2103 (49.32)	3940 (49.86)		
Having regular exercises					
No exercise	2245(61.71)	2596 (60.88)	4841 (61.26)	1.028	0.598
Less than exercises	686(18.86)	842 (19.75)	1528 (19.34)		
egular exercises	707(19.43)	826 (19.37)	1533 (19.40)		
Chronic diseases(counts)					
0	1267(34.83)	1322 (31.00)	2589 (32.76)	13.892	0.001
01-Feb	1820(50.03)	2226 (52.20)	4046 (51.20)		
Mar-14	551(15.15)	716 (16.79)	1267 (16.03)		
WC	84.22 ± 9.49	84.99 ± 10.05	84.63 ± 9.80	-3.487	< 0.001
BMI	22.72 ± 3.48	23.78 ± 3.92	23.29 ± 3.76	-12.738	< 0.001
WHtR	0.51 ± 0.06	0.56 ± 0.07	0.54 ± 0.06	-31.109	< 0.001
VAI	3.49 ± 3.35	5.47 ± 4.77	4.56 ± 4.29	-21.566	< 0.001
ABSI	0.08 ± 0.01	0.08 ± 0.01	0.08 ± 0.01	-9.591	< 0.001
BRI	3.69 ± 1.10	4.57 ± 1.41	4.16 ± 1.35	-31.091	< 0.001
LAP	27.05 ± 26.61	39.83 ± 30.87	33.95 ± 29.67	-19.758	< 0.001
CI	1.27 ± 0.08	1.30 ± 0.10	1.28 ± 0.09	-14.182	< 0.001
CVAI	91.47 ± 45.25	102.64 ± 42.06	97.49 ± 43.91	-11.295	< 0.001
TyG index	8.50 ± 0.56	8.60 ± 0.54	8.56 ± 0.55	-8.467	< 0.001
TyG-BMI	193.63 ± 35.46	205.10 ± 39.08	199.82 ± 37.89	-13.677	< 0.001
TyG-WC	717.41 ± 106.25	732.57 ± 108.26	725.59 ± 107.60	-6.26	< 0.001
TyG -WHtR	4.38 ± 0.62	4.80 ± 0.71	4.61 ± 0.70	-27.746	< 0.001

WC, waist circumference; BMI, body mass index; WHtR, waist to height ratio;VAI, visceral adiposity index; ABSI, A body shape index; BRI, body roundness index; LAP, lipid accumulation product; CVAI, cardio-ankle vascular index; CI, conicity index; TyG, triglyceride and glucose index; TyG-BMI, TyG related to BMI; TyG-WC, TyG related to WC; TyG-WHtR, TyG related to WHtR.

**Table 2** presents the baseline characteristics of T2DM and non-diabetic subjects by gender. Based on the findings, the percentage of women with T2DM was significantly higher (9.15% versus 9.02% for males). There were significant differences in WC, BMI, WHtR, VAI, ABSI, BRI, LAP, CI, CVAI, TyG, TyG-BMI, TyG-WC, and TyG-WHtR in men with T2DM (*P<* 0.05); There were significant differences in age, current residence, chronic illness, WC, BMI, WHtR, VAI, ABSI, BRI, LAP, CI, CVAI, CVAI, TyG-BMI, TyG-WC, and TyG-WHtR in women with T2DM (*P<* 0.05). There were no significant differences among T2DM subgroups, male or female, in terms of educational background, marital status, current smoking, alcohol drinking, taking activities, and having regular exercises (*P* > 0.05).

**Table 2:** Baseline characteristics of the study participants with and without T2DM by sex.

Variables	Male (N=3638)		*χ2*	*P*	Female (N=4264)		*χ2*	*P*
N (%)	With T2DM N (%)	Without T2DM N (%)			With T2DM N (%)	Without T2DM N (%)		
N	328 (9.02)	3310 (90.98)			390 (9.15)	3874 (90.85)		
Age(years)								
45-54	85 (25.91)	1022 (30.88)	3.745	0.290	126(32.31)	1547 (39.93)	15.792	0.001
55-64	140 (42.68)	1280 (38.67)			142 (36.41)	1420 (36.65)		
65-74	75 (22.87)	739 (22.33)			90 (23.08)	626 (16.16)		
≥75	28 (8.54)	269(8.13)			32 (8.21)	281 (7.25)		
Education								
Illiterate	38 (11.59)	463 (13.99)	3.632	0.304	177 (45.38)	1630 (42.08)	6.344	0.096
Less than elementary school	255 (77.74)	2414 (72.93)			198 (50.77)	1975 (50.98)		
High school	22 (6.71)	284 (8.58)			10 (2.56)	202 (5.21)		
Above vocational school	13 (3.96)	149 (4.50)			5 (1.28)	67 (1.73)		
Marital status								
Single	32 (9.76)	305 (9.21)	0.104	0.747	68(17.44)	577 (14.89)	1.783	0.182
Married	296 (90.24)	3005 (90.79)			322 (82.56)	3297 (85.11)		
Current residence								
Rural	300 (91.46)	3063 (92.54)	0.493	0.483	373 (95.64)	3595 (92.80)	4.433	0.035
Urban	28 (8.54)	247 (7.46)			17 (4.36)	279 (7.20)		
Current smoking								
No	75 (22.87)	818 (24.71)	0.647	0.724	359 (92.05)	3578 (92.36)	0.638	0.727
Former smoke	52 (15.85)	533 (16.10)			9 (2.31)	68 (1.76)		
Current smoke	201 (61.28)	1959 (59.18)			22 (5.64)	228 (5.89)		
Alcohol drinking								
No	156 (47.56)	1436 (43.38)	2.117	0.347	352 (90.26)	3378 (87.20)	3.036	0.219
Less than once a month	34 (10.37)	369 (11.15)			15 (3.85)	200 (5.16)		
More than once a month	138 (42.07)	1505 (45.47)			23 (5.90)	296 (7.64)		
Taking activities								
No	158 (48.17)	1643 (49.64)	0.257	0.612	205 (52.56)	1956 (50.49)	0.610	0.435
Yes	170 (51.83)	1667 (50.36)			185 (47.44)	1918 (49.51)		
Having regular exercises								
No exercise	199 (60.67)	2046 (61.81)	0.173	0.917	231 (59.23)	2365 (61.05)	1.801	0.406
Less than exercises	64 (19.51)	622 (18.79)			87 (22.31)	755 (19.49)		
Regular exercises	65 (19.82)	642 (19.40)			72 (18.46)	754 (19.46)		
Chronic diseases(counts)								
0	106 (32.32)	1161 (35.08)	4.123	0.127	91 (23.33)	1231 (31.78)	13.312	0.001
1-2	160 (48.78)	1660 (50.15)			218 (55.90)	2008 (51.83)		
3-14	62 (18.90)	489 (14.77)			81 (20.77)	635 (16.39)		
WC	87.06 ± 10.68	83.94 ± 9.32	-5.113	< 0.001	89.61 ± 10.43	84.52 ± 9.89	-9.631	< 0.001
BMI	23.70 ± 4.28	22.62 ± 3.38	-4.417	< 0.001	25.31 ± 3.96	23.63 ± 3.89	-8.107	< 0.001
WHtR	0.53 ± 0.06	0.51 ± 0.05	-5.489	< 0.001	0.59 ± 0.07	0.55 ± 0.06	-9.528	< 0.001
VAI	4.39 ± 4.34	3.41 ± 3.22	-4.010	< 0.001	6.79 ± 5.28	5.34 ± 4.70	-5.201	< 0.001
ABSI	0.08 ± 0.01	0.08 ± 0.01	-2.120	0.034	0.08 ± 0.01	0.08 ± 0.01	-2.868	0.004
BRI	4.06 ± 1.28	3.65 ± 1.07	-5.559	< 0.001	5.22 ± 1.51	4.50 ± 1.39	-9.613	< 0.001
LAP	36.06 ± 37.00	26.16 ± 25.18	-4.738	< 0.001	52.43 ± 35.78	38.56 ± 30.05	-7.395	< 0.001
CI	1.29 ± 0.09	1.27 ± 0.08	-4.016	< 0.001	1.32 ± 0.09	1.29 ± 0.10	-5.943	< 0.001
CVAI	106.66 ± 51.51	89.96 ± 44.31	-5.669	< 0.001	124.29 ± 40.84	100.45 ± 41.56	-10.813	< 0.001
TyG index	8.64 ± 0.60	8.49 ± 0.55	-4.380	< 0.001	8.79 ± 0.52	8.58 ± 0.54	-7.249	< 0.001
TyG-BMI	205.33 ± 42.58	192.47 ± 34.47	-5.299	< 0.001	222.74 ± 38.60	203.33 ± 38.69	-9.447	< 0.001
TyG-WC	754.21 ± 122.30	713.76 ± 103.83	-5.787	< 0.001	788.49 ± 107.94	726.94 ± 106.70	-10.847	< 0.001
TyG -WHtR	4.61 ± 0.72	4.36 ± 0.61	-6.127	< 0.001	5.16 ± 0.69	4.76 ± 0.70	-10.813	< 0.001

WC, waist circumference; BMI, body mass index; WHtR, waist to height ratio; VAI, visceral adiposity index; ABSI, A body shape index; BRI, body roundness index; LAP, lipid accumulation product; CVAI, cardio-ankle vascular index; CI, conicity index; TyG, triglyceride and glucose index; TyG-BMI, TyG related to BMI; TyG-WC, TyG related to WC; TyG-WHtR, TyG related to WHtR.

**Figure 1:**
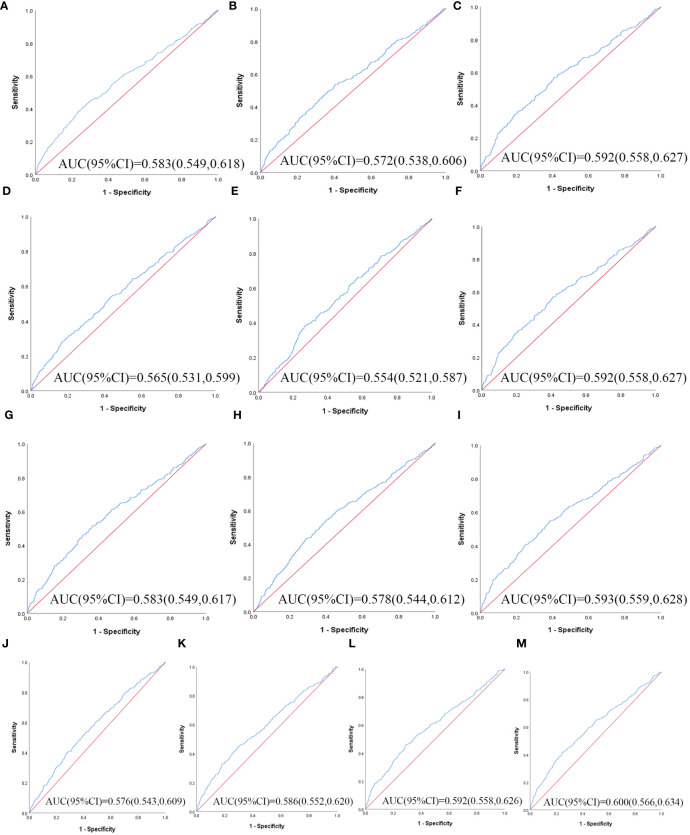
The ROC curves of each indicator in the prediction of T2DM risk in males. **(A)** WC, **(B)** BMI, **(C)** WHtR, **(D)** VAI, **(E)** ABSI, **(F)** BRI, **(G)** LAP, **(H)** CI, **(I)** CVAI, **(J)** TyG-index, **(K)** TyG-BMI, **(L)** TyG-WC, **(M)** TyG-WHtR.

**Figure 2:**
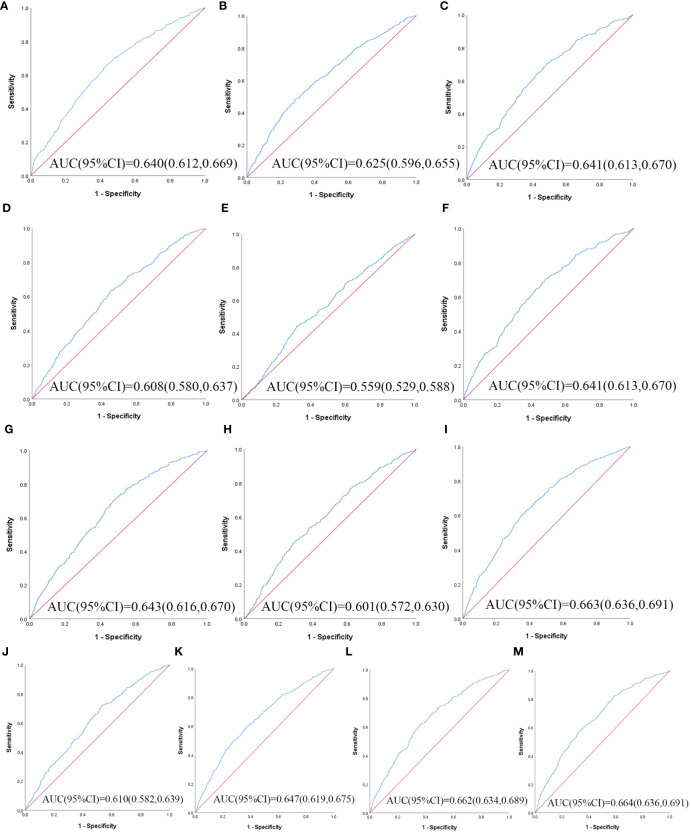
The ROC curves of each indicator in the prediction of T2DM risk in females. **(A)** WC, **(B)** BMI, **(C)** WHtR, **(D)** VAI, **(E)** ABSI, **(F)** BRI, **(G)** LAP, **(H)** CI, **(I)** CVAI, **(J)** TyG-index, **(K)** TyG-BMI, **(L)** TyG-WC, **(M)** TyG-WHtR.

**Table 3** illustrates the evaluation of the prediction using ROC curves and AUC. The ROC curves of each parameter for predicting the risk of T2DM are presented in **Figures 1**, **2**. In men, the TyG -WHtR was the best predictor of T2DM in the middle-aged and elderly male population (AUC=0.600, SE=0.017, 95% CI=0.566-0.634, and optimal cut-off=4.769). Meanwhile, it can be observed from the table that WC (AUC = 0.583, S E = 0.018, 95% CI = 0.549-0.618, and optimal cut-off =88.850) and LAP (AUC = 0.583, S E = 0.017, 95% CI = 0.549-0.617, and optimal cut-off =28.333) had similar predictive values. Similarly, the predictive values were similar for the WHtR (AUC = 0.592, SE = 0.017, 95% CI =0.558-0.627, and optimal cut-off = 0.520), BRI (AUC = 0.592, SE = 0.017, 95% CI =0.558-0.627, and optimal cut-off =3.739) and TyG-WC (AUC = 0.592, SE = 0.017, 95% CI =0.558-0.626, and optimal cut-off = 765.677). In addition, in female patients, TyG -WHtR was also the best predictor of T2DM (AUC=0.664, SE=0.015, 95%CI 0.636-0.691, and optimal cut-off=5.031). Similarly, the predictive values were similar for the WHtR (AUC = 0.641, SE = 0.014, 95% CI =0.613-0.670, and optimal cut-off = 0.553), BRI (AUC = 0.641, SE = 0.014, 95% CI =0.613-0.670, and optimal cut-off =4.416). There was a statistical difference in all the indexes (*P*< 0.05). The results showed that the AUC of these 13 indexes was above 0.5, suggesting that they could predict the development of T2DM.

**Table 3:** Cut-off between area under curve, sensitivity, and specificity for obesity- and lipid-related indices to detect T2DM by sex.

N=7902	WC	BMI	WHtR	VAI	ABSI	BRI	LAP	CI	CVAI	TyG index	TyG-BMI	TyG-WC	TyG -WHtR
Male													
Area under curve	0.583	0.572	0.592	0.565	0.554	0.592	0.583	0.578	0.593	0.576	0.586	0.592	0.600
Std. Error	0.018	0.017	0.017	0.017	0.017	0.017	0.017	0.017	0.018	0.017	0.017	0.017	0.017
95%CI	0.549,0.618	0.538,0.606	0.558,0.627	0.531,0.599	0.521,0.587	0.558,0.627	0.549,0.617	0.544,0.612	0.559,0.628	0.543,0.609	0.552,0.620	0.558,0.626	0.566,0.634
* P*-value	< 0.001	< 0.001	< 0.001	< 0.001	0.001	< 0.001	< 0.001	< 0.001	< 0.001	< 0.001	< 0.001	< 0.001	< 0.001
Optimal cutoffs	88.850	23.082	0.520	4.573	0.085	3.739	28.333	1.307	99.429	8.762	207.797	765.677	4.769
J-Youden	0.151	0.135	0.160	0.113	0.114	0.160	0.151	0.145	0.166	0.126	0.154	0.171	0.173
Sensitivity (%)	44.21%	53.70%	57.01%	31.70%	38.41%	57.01%	46.34%	42.99%	55.18%	39.94%	44.51%	46.04%	41.46%
Specificity (%)	70.85%	59.80%	59.03%	79.60%	72.99%	59.03%	68.79%	71.45%	61.39%	72.66%	70.91%	71.06%	75.80%
(+) Likelihood ratio	1.516	1.336	1.392	1.554	1.422	1.392	1.485	1.506	1.429	1.461	1.530	1.591	1.713
(-) Likelihood ratio	0.788	0.774	0.728	0.858	0.844	0.728	0.780	0.798	0.730	0.827	0.783	0.759	0.772
Female													
Area under curve	0.640	0.625	0.641	0.608	0.559	0.641	0.643	0.601	0.663	0.610	0.647	0.662	0.664
Std. Error	0.015	0.015	0.014	0.015	0.015	0.014	0.014	0.015	0.014	0.014	0.015	0.014	0.014
95%CI	0.612,0.669	0.596,0.655	0.613,0.670	0.580,0.637	0.529,0.588	0.613,0.670	0.616,0.670	0.572,0.630	0.636,0.691	0.582,0.639	0.619,0.675	0.634,0.689	0.636,0.691
* P*-value	< 0.001	< 0.001	< 0.001	< 0.001	< 0.001	< 0.001	< 0.001	< 0.001	< 0.001	< 0.001	< 0.001	< 0.001	< 0.001
Optimal cutoffs	84.950	25.422	0.553	4.277	0.086	4.416	30.835	1.341	114.005	8.516	212.290	759.606	5.031
J-Youden	0.224	0.198	0.223	0.183	0.123	0.223	0.234	0.163	0.253	0.191	0.228	0.256	0.248
Sensitivity (%)	70.30%	48.72%	70.51%	63.33%	44.40%	70.51%	72.05%	44.90%	60.51%	72.10%	59.70%	61.28%	58.72%
Specificity (%)	52.10%	71.09%	51.83%	55.01%	67.90%	51.83%	51.34%	71.40%	64.79%	47.00%	63.10%	64.27%	66.06%
(+) Likelihood ratio	1.468	1.685	1.464	1.408	1.383	1.464	1.481	1.570	1.719	1.360	1.618	1.715	1.730
(-) Likelihood ratio	0.570	0.721	0.569	0.667	0.819	0.569	0.544	0.772	0.609	0.594	0.639	0.602	0.625

WC, waist circumference; BMI, body mass index; WHtR, waist to height ratio; VAI, visceral adiposity index; ABSI, A body shape index; BRI, body roundness index; LAP, lipid accumulation product; CVAI, cardio-ankle vascular index; CI, conicity index; TyG, triglyceride and glucose index; TyG-BMI, TyG related to BMI; TyG-WC, TyG related to WC; TyG-WHtR, TyG related to WHtR.

**Figure 3:**
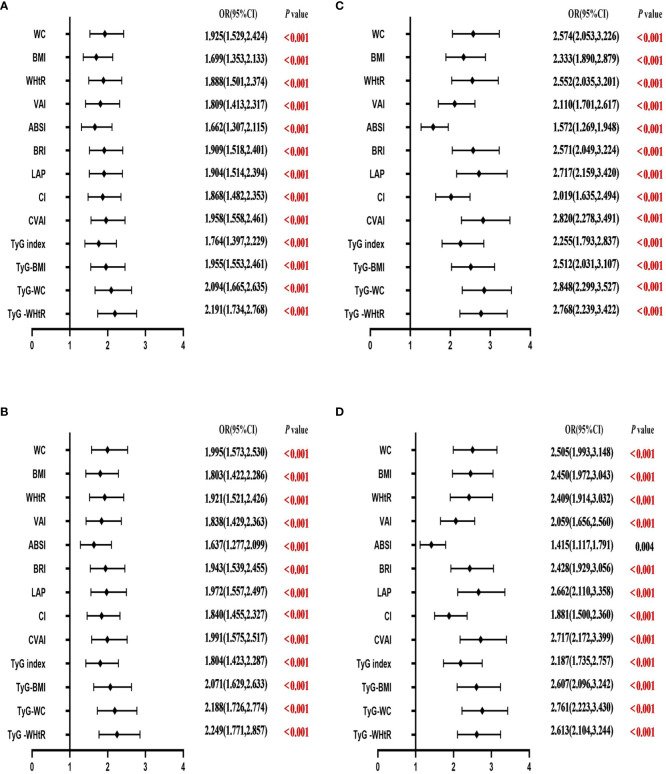
Forest diagram of OR before and after adjustment of confounding factors for males and females. **(A)** Male unadjusted; **(B)** male adjusted; **(C)** female unadjusted; **(D)** female adjusted. Adjusted OR: Adjusted for age, educational levels, marital status, live place, current smoking, alcohol drinking, activities, exercises, and chronic diseases.

**Table 4** shows the associations of obesity- and lipid-related indices with T2DM. In this survey, 13 obesity and lipid indices were converted into two categories according to the figures in **Table 3**. **Table 4** is based on the transformed variables. In general, the greater the OR, the higher the risk. After adjusting for factors such as age, education, marital status, current residence, current smoking, alcohol drinking, taking activities, having regular exercises, and chronic diseases, both men’s and women’s odds of developing T2DM gradually increased with increasing obesity and lipid measurement units. After adjusting for all covariates, each unit rise in TyG -WHtR, for example, was related to a 2.249-fold (95% CI=1.771-2.857) increase in the likelihood of developing T2DM in males. Each unit increase in TyG-WC was linked to a 2.761-fold (95% CI=2.223-3.430) increase in the likelihood of developing T2DM in females. Among the 13 indicators, the correlation between ABSI and T2DM was weakest in males (OR=1.637, 95% CI: 1.277-2.099) and females (OR=1.415, 95% CI: 1.117-1.791). After adjusting for confounding factors, all indicators were statistically significant (*P*< 0.05). **Figure 3** illustrates the forest plot or values before and after adjustment for the confounding factors for men and women.

**Table 4:** Associations of obesity- and lipid-related indices with T2DM and its components.

T2DM	WC	BMI	WHtR	VAI	ABSI	BRI	LAP	CI	CVAI	TyG index	TyG-BMI	TyG-WC	TyG -WHtR
Male													
Unadjusted OR (95% CI)	1.925(1.529,2.424) ^**^	1.699(1.353,2.133) ^**^	1.888(1.501,2.374) ^**^	1.809(1.413,2.317) ^**^	1.662(1.307,2.115) ^**^	1.909(1.518,2.401) ^**^	1.904(1.514,2.394)^**^	1.868(1.482,2.353) ^**^	1.958(1.558,2.461)^**^	1.764(1.397,2.229) ^**^	1.955(1.553,2.461)^**^	2.094(1.665,2.635) ^**^	2.191(1.734,2.768) ^**^
*P* value	< 0.001	< 0.001	< 0.001	< 0.001	< 0.001	< 0.001	< 0.001	< 0.001	< 0.001	< 0.001	< 0.001	< 0.001	< 0.001
Adjusted OR (95% CI)	1.995(1.573,2.530) ^**^	1.803(1.422,2.286) ^**^	1.921(1.521,2.426) ^**^	1.838(1.429,2.363) ^**^	1.637(1.277,2.099) ^**^	1.943(1.539,2.455) ^**^	1.972(1.557,2.497) ^**^	1.840(1.455,2.327) ^**^	1.991(1.575,2.517) ^**^	1.804(1.423,2.287) ^**^	2.071(1.629,2.633) ^**^	2.188(1.726,2.774) ^**^	2.249(1.771,2.857)^**^
*P* value	< 0.001	< 0.001	< 0.001	< 0.001	< 0.001	< 0.001	< 0.001	< 0.001	< 0.001	< 0.001	< 0.001	< 0.001	< 0.001
Female													
Unadjusted OR (95% CI)	2.574(2.053,3.226) ^**^	2.333(1.890,2.879) ^**^	2.552(2.035,3.201) ^**^	2.110(1.701,2.617) ^**^	1.572(1.269,1.948) ^**^	2.571(2.049,3.224) ^**^	2.717(2.159,3.420) ^**^	2.019(1.635,2.494) ^**^	2.820(2.278,3.491) ^**^	2.255(1.793,2.837) ^**^	2.512(2.031,3.107) ^**^	2.848(2.299,3.527) ^**^	2.768(2.239,3.422) ^**^
*P* value	< 0.001	< 0.001	< 0.001	< 0.001	< 0.001	< 0.001	< 0.001	< 0.001	< 0.001	< 0.001	< 0.001	< 0.001	< 0.001
Adjusted OR (95% CI)	2.505(1.993,3.148) ^**^	2.450(1.972,3.043)^**^	2.409(1.914,3.032) ^**^	2.059(1.656,2.560) ^**^	1.415(1.117,1.791) ^*^	2.428(1.929,3.056) ^**^	2.662(2.110,3.358) ^**^	1.881(1.500,2.360) ^**^	2.717(2.172,3.399)^**^	2.187(1.735,2.757) ^**^	2.607(2.096,3.242) ^**^	2.761(2.223,3.430) ^**^	2.613(2.104,3.244) ^**^
*P* value	< 0.001	< 0.001	< 0.001	< 0.001	0.004	< 0.001	< 0.001	< 0.001	< 0.001	< 0.001	< 0.001	< 0.001	< 0.001

WC, waist circumference; BMI, body mass index; WHtR, waist to height ratio; VAI, visceral adiposity index; ABSI, A body shape index; BRI, body roundness index; LAP, lipid accumulation product; CVAI, cardio-ankle vascular index; CI, conicity index; TyG, triglyceride and glucose index; TyG-BMI, TyG related to BMI; TyG-WC, TyG related to WC; TyG-WHtR, TyG related to WHtR.

Odds ratios were adjusted for age, educational levels, marital status, live place, current smoking, alcohol drinking, activities, exercises, chronic diseases.

^*^P<0.05, ^**^P<0.001.

## Discussion

T2DM has been identified as a major public health problem with significant implications for people’s lives and health spending. A significant risk factor for T2DM is obesity. Finding a quick and easy way to test for diabetes can therefore serve as a foundation for an early diagnosis of T2DM. This study evaluated the predictive power of 13 obesity- and lipid-related indices in identifying the risk of T2DM in middle-aged and old adults in China. In our study, a total of 7902 people participated in the study. After adjusting for population factors in our population-based cohort analysis, we found a statistically significant relationship between obesity and lipid-related indices and T2DM.

This study revealed that 13 obesity and lipid-related indicators are all related to the risk of T2DM. Similar to other studies, all 13 indicators play an important role in different populations (43–45). After adjusting for multiple covariates, as the 13 indicators increase, the prevalence of T2DM increases. Among them, TyG-WHtR and CVAI have a stronger correlation with the prevalence of T2DM.

As far as we know, insulin resistance (IR) and islet β-cell dysfunction are the main pathophysiology of T2DM (46–48). A great deal of hormones and cytokines are produced in adipose and adipose tissue, which are important in glucose metabolism and lipid metabolism (49). A characteristic of several metabolic diseases, including hyperglycemia and hypertriglyceridemia, is IR (50). Ahn et al. have shown that the TyG index is composed of factors related to fat and blood sugar, and it is a reliable indicator that has a good reflection of human IR (10). Studies have shown that the TyG index can be a cheap and reliable indicator for the diagnosis of IR, which is important for the early diagnosis of T2DM high-risk people (51). The TyG index, a new measure based on TG and FBG, has been shown to be a useful predictor of metabolic disorders and T2DM (52–54). Several studies have suggested that TyG index is significantly linked to the chance of acquiring T2DM in Singapore (55), Japan (56), Korea (57), and Thailand (58). Ferreira, J.R.S (59). has shown that the TyG-related factors are a useful tool to predict metabolic syndrome. So far, the majority of studies (60–62) have also demonstrated a strong relationship between the TyG-related factors and pre-diabetes/diabetes.

In fact, factors related to TyG played a far greater role in this study than other factors. In our study, TyG-related factors such as TyG-WHtR, TyG-BMI, and TyG-WC can provide a broader basis for obesity- and lipid-related indices to estimate T2DM. TyG-WHtR (AUC=0.600 in males and 0.664 in females) had greater efficacy in predicting T2DM symptoms than the TyG index alone. Many earlier several studies share similar views to our findings (63, 64). As the TyG index is a reliable and alternate indicator of IR, it may be used to assess the risk of T2DM. It is easy to get and calculate it in the clinic or large-scale epidemiological survey.

In addition, our study showed that women have a higher risk of developing T2DM than men. This may be because women lose more height than men and have more subcutaneous fat storage. One study showed that increased visceral fat levels were associated with about a threefold increased risk of T2DM in women, and a modest 20% increased risk in men (65). The central distribution of adipose tissue has a greater impact on the incidence of non-insulin-dependent T2DM in women than in men and may lead to an increased risk of T2DM (65, 66). Together, consistent with our study, older women have a higher risk of new-onset T2DM than men and are more sensitive to predicting the prevalence of T2DM in women with a higher area under the curve for most indicators.

Interestingly, in our study, women were 2.761 times more likely to develop T2DM for each unit increase in TyG-WC (95% CI=2.223-3.430), and a similar study reported a stronger correlation between visceral adiposity and serum TG in women than in men (67). This may be because women generally show higher hepatocyte lipids on an empty stomach and after a glucose and lipid load (68). Several studies (64, 69) have jointly shown that TyG-WC can be used as the main monitoring parameter for diabetes screening and clinical assessment/prediction of diabetes risk in the population, which also has certain reference value for our research.

BMI and WC are safe, easy, and cheap tools to evaluate a person’s health status and make a rough estimate of the risk of obesity, including T2DM. In a retrospective study of 41, 242 people aged 45 or older, the relationship between BMI and WC and T2DM was higher than that of WHtR or TyG index (70). Qiwei Ge et al. (71) also found that WC was the best predictor of T2DM in elderly men. However, these studies’ indicators differed from those in our analysis. In our research, BMI and WC were relatively weak in predicting T2DM. A Japanese cohort study (72) showed that BRI was superior to BMI and WC in predicting T2DM. Moreover, it is noteworthy that Jayedi et al.’s recent meta-analysis of 216 cohort studies, which was published in the BMJ, demonstrated that WHtR was more closely linked to T2DM in routine assessments than WC, waist-to-hip ratio, and BMI (73). With some reports demonstrate that WHtR had higher predictive power than WC and BMI (74, 75). The cut-off values for BMI and WC are greatly impacted by gender differences, and both BMI and WC have various limits (76). Perhaps in the future, BMI can be combined with WHtR and WC, which can improve the risk phenotype of T2DM and screen diabetic patients.

VAI is the visceral obesity index (77), which was often used in the past in Caucasians, and the correlation with the area of adipose tissue in the Chinese body is low, and the difference may be related to the different distribution of adipose tissue in the body of Caucasians and Asians, and Asians may be more prone to have visceral fat accumulation, which may be related to different lifestyles. Therefore, in order to find an index that better represents the characteristics of body fat distribution and has a higher predictive value, CVAI is a new index for the evaluation of visceral adipose. Research indicates that among Chinese individuals, CVAI positively correlates with the risk of T2DM (23, 24, 78). The Japanese study indicated a significant association between CVAI and T2DM risk, which further confirmed the efficacy of CVAI in Asia, and was superior to BMI and WC in predicting T2DM. This could be due to the fact that CVAI is a composite of age, BMI, WC, and blood lipids. Therefore, it is superior to a single index. In our study, the AUC of CVAI was smaller than that of TyG-WHtR in middle-aged and older adults in both males (AUC = 0.593, Std. Error = 0.018, 95% CI = 0.559–0.628, and optimal cutoff value = 99.429) and female (AUC = 0.663, Std. Error = 0.014, 95% CI = 0.636–0.691, and optimal cutoff value = 114.005), making CVAI the second-best indicator after TyG-WHtR in this group. A 5-year prospective study in China showed that it was superior to VAI and anthropometric indicators in assessing metabolic risk (79). Similarly, Wei In review*er al.* also found that CVAI outperformed WC, BMI, and ABSI in T2DM screening among Chinese adults (22).

Similarly, LAP is a good predictor of metabolic synthesis. LAP was found to be a strong predictor of insulin resistance (80, 81), indicating a significant relationship between LAP and T2DM. The greatest component in this study’s ROC analysis of obesity- and lipid-associated measurements for females was the AUC of LAP (AUC = 0.643, Std. Error = 0.014, 95% CI = 0.616-0.670, and optimal cut-off = 30.835), which was excepted for the TyG related factors and CVAI.

In this study, 13 obesity- and lipid-related indices were transformed into two categories based on the optimum cut-off point in **Table 3**. **Table 4** is based on the transformed variables. In general, a higher OR indicates a greater risk factor. In **Table 4**, the ABSI OR was significantly lower than the other 12 indices (OR=1.637 in males and 1.415 in females), after adjustment for all confounding factors. According to ROC analysis, the AUC value of ABSI is lower than the other 12 indices (AUC=0.554 in males and 0.559 in females). Consistent with our research results, Chang et al. also found that in terms of predicting the existence of T2DM among the rural population in Northeast China, compared with BRI, ABSI has the weakest predictive ability (82).

In this study, the relationship between obesity and lipid-associated index and T2DM was discussed. These straightforward and easily measured indicators can assist middle-aged and elderly individuals in implementing early intervention measures, such as lifestyle modifications (balanced diet and appropriate physical activity), to prevent the occurrence of T2DM. Because T2DM is a chronic disease, early detection of potential risk factors and maintaining a healthy lifestyle are key to preventing it. In the field of public health, the results can be used as a reference for clinical practice, public health consultation, and for identifying high-risk groups for prevention.

### Strengths and limitations of the study

Our research is based on a nationally representative longitudinal dataset. As far as we know, aging-related diseases are rapidly expanding among the elderly in China, and this is the first cohort study to evaluate the association between 13 obesity and lipid-related indicators and the prevalence of T2DM among the elderly in China. Secondly, the measurement methods of most indicators are simple to operate and can be popularized in clinical practice. In addition, this study included 7902 people aged 45 years and older, a large sample size, which provides a scientific basis and theoretical basis for the prevention and treatment of T2DM. The study does have some limitations. First of all, this study focuses on middle-aged and elderly people in China, and it is difficult to apply the conclusions of this study to elderly people in other countries when there are differences between the East and the West. Second, although we adjusted for multiple influencing factors such as education level, smoking status, drinking status, and chronic diseases, undetected residual factors may alter the relationship between obesity and T2DM or hinder its development.

## Conclusions

In conclusion, our findings revealed that T2DM was connected to overall obesity- and lipid-related indicators. Additionally, the TyG-WHtR is the most accurate marker for detecting T2DM in both males and females. CVAI was a good predictor of T2DM in males and females. Our findings highlight the importance of increasing knowledge about T2DM and improving health care.

## Data availability statement

The datasets presented in this study can be found in online repositories. The names of the repository/repositories and accession number(s) can be found in the article/supplementary material.

## Ethics statement

All data are openly published as microdata at http://opendata.pku.edu.cn/dataverse/CHARLS with no direct contact with all participants. Approval for this study was given by the medical ethics committee of Wannan medical college (approval number 2021–3).

## Author contributions

YW: Writing – original draft, Writing – review & editing. XZ: Writing – review & editing. YQL: Writing – review & editing. JG: Writing – review & editing. YM: Writing – review & editing. XY: Writing – review & editing. HL: Writing – review & editing. L-LG: Writing – review & editing. JLL: Writing – review & editing. YXL: Writing – review & editing. XL: Writing – review & editing. LS: Writing – review & editing. LY: Writing – review & editing. TY: Writing – review & editing. CW: Writing – review & editing. DZ: Writing – review & editing. JL: Writing – review & editing. ML: Writing – review & editing. YH: Writing – review & editing. LZ: Methodology, Writing – review & editing.
